# Relationship between mortality and use of sodium bicarbonate at the time of dialysis initiation: a prospective observational study

**DOI:** 10.1186/s12882-021-02330-0

**Published:** 2021-04-06

**Authors:** Hikaru Morooka, Junichiro Yamamoto, Akihito Tanaka, Daijo Inaguma, Shoichi Maruyama

**Affiliations:** 1 Division of Nephrology, Nagoya University Hospital, Tsurumaicho, 65, Showa Ward, Nagoya, Aichi Japan; 2 Division of Nephrology, Tsushima City Hospital, Tsushima, Japan; 3 Division of Internal Medicine, Fujita Health University Bantane Hospital, Nagoya, Japan; 4 Division of Nephrology, Nagoya University Graduate School of Medicine, Nagoya, Japan

**Keywords:** Sodium bicarbonate, Chronic kidney disease, Dialysis, Mortality

## Abstract

**Background:**

Patients with chronic kidney disease often experience metabolic acidosis. Whether oral sodium bicarbonate can reduce mortality in patients with metabolic acidosis has been debated for years. Hence, this study was conducted to evaluate the utility of sodium bicarbonate in patients who will undergo dialysis therapy. In this study, we investigated the effect of oral sodium bicarbonate therapy on mortality in patients with end-stage kidney disease (ESKD) initiated on dialysis therapy.

**Methods:**

We conducted an observational study of patients when they started dialysis therapy. There were 17 centres participating in the Aichi Cohort Study of Prognosis in Patients Newly Initiated into Dialysis. Data were available on patients’ sex, age, use of sodium bicarbonate, drug history, medical history, vital data, and laboratory data. We investigated whether patients on oral sodium bicarbonate for more than three months before dialysis initiation had a better prognosis than those without sodium bicarbonate therapy. The primary outcome was defined as all-cause mortality.

**Results:**

The study included 1524 patients with chronic kidney disease who initiated dialysis between October 2011 and September 2013. Among them, 1030 were men and 492 women, with a mean age of 67.5 ± 13.1 years. Of these, 677 used sodium bicarbonate and 845 did not; 13.6% of the patients in the former group and 21.2% of those in the latter group died by March 2015 (*p* <  0.001). Even after adjusting for various factors, the use of sodium bicarbonate independently reduced mortality (*p* <  0.001).

**Conclusions:**

The use of oral sodium bicarbonate at the time of dialysis initiation significantly reduced all-cause mortality in patients undergoing dialysis therapy.

**Supplementary Information:**

The online version contains supplementary material available at 10.1186/s12882-021-02330-0.

## Introduction

Chronic kidney disease (CKD) affects 8–16% of the population worldwide and is often under-recognised by both patients and clinicians [1]. CKD is the 16th leading cause of years of life lost worldwide [1]. In patients with CKD, the risk of death increases as kidney function worsens and such deaths are largely attributable to cardiovascular disease, although cancer incidence and mortality are also increased in these patients [2, 3]. Moreover, patients with CKD often experience metabolic acidosis resulting from a fundamental disturbance of acid-base balance. As serum bicarbonate (HCO_3_^−^) is below the normal range in patients with CKD, generally considered as 22–29 mEq/L, the risk of progression to end-stage kidney disease (ESKD) or death is higher compared to those with CKD but without metabolic acidosis [4–10]. The treatment for metabolic acidosis in CKD is oral alkali supplementation, which has been shown to slow CKD progression [11–13]. When the bicarbonate level is below 22 mmol/L, prescription of sodium bicarbonate is recommended [14]. However, the available evidence only provides general guidance for clinicians and has substantial variability in clinical practice [15].

Oral sodium bicarbonate has been used for decades to counteract metabolic acidosis [3]. Oral sodium bicarbonate may have a residual renal function-preserving effect in peritoneal dialysis patients [16]. Moreover, bicarbonate supplementation modestly improved renal function and serum bicarbonate levels compared to placebo or conventional CKD management in non-haemodialysis dependent CKD patients [17]. In contrast, there are concerns that the sodium content might increase blood pressure or circulatory overload, leading to the deterioration of heart failure [3]. Nonetheless, it is unknown how oral bicarbonate supplementation can affect patients who start dialysis therapy both positively and negatively.

In the present study, we aimed to investigate the effect of oral sodium bicarbonate therapy on mortality in patients with ESKD who were initiated on dialysis therapy. Our study is novel and significant because we focused on the efficacy of oral sodium bicarbonate therapy in patients before dialysis therapy initiation.

## Patients and methods

### Patient registration and data collection

**Fig. 1:**
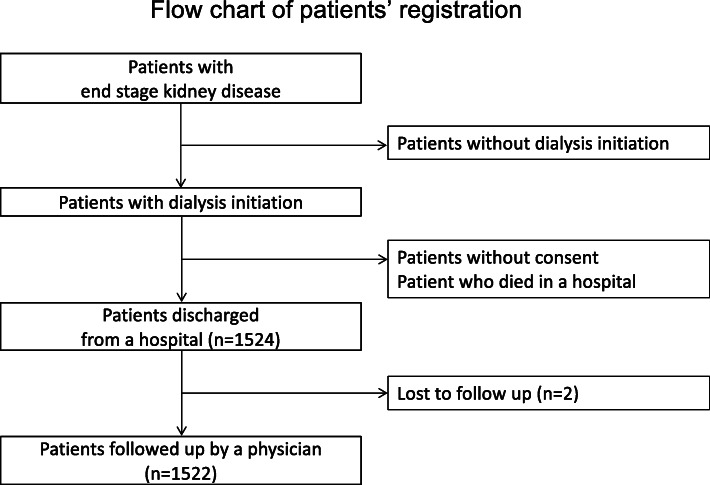
Flow chart showing the process of patient registration. Only patients who became stable and were discharged or transferred from the hospital with consent were included. Patients who were not discharged and died in the hospital were excluded

Data from the Aichi Cohort Study of Prognosis in Patients Newly Initiated into Dialysis [18, 19] were used in this prospective multicentre study. Participants were patients who commenced dialysis between October 2011 and September 2013 at 17 Japanese institutions. This study was approved by the Ethics Committee of the Institutional Review Board of Nagoya University Hospital (approval number: 1335), and all patients provided written informed consent. First, we screened all the patients with ESKD who were undergoing dialysis. Only patients whose condition became stable and were discharged or transferred from the hospital were included in this study. Patients who were not discharged and died in the hospital were excluded from the study (Fig. 1). Data regarding patients’ background, medical history, comorbidities, medications, and laboratory data during the period of dialysis initiation were collected. Each physician judged whether the patients with ESKD had heart failure symptoms at the initiation of dialysis therapy based on the Framingham Heart Study [20]. The use of sodium bicarbonate was assigned to those who were on oral sodium bicarbonate for more than 3 months before the dialysis initiation. The dosage and total duration of the oral sodium bicarbonate were unknown. Serologic data were obtained mainly from venous at the first dialysis session, just before dialysis initiation. Bicarbonate levels were analysed by a blood gas analyser. Patients were followed till the end of March 2015.

### Mortality

The patients were divided into 2 groups: those with and without the use of oral sodium bicarbonate. The primary endpoint was all-cause mortality. Causes of death were recorded to the maximum extent possible. The occurrence of death was investigated via survey slips sent to the dialysis facilities at the end of March 2015, until we could finally get the replies.

We compared the outcomes and hazard ratios (HRs) between the two groups.

### Statistics

Baseline characteristics were presented descriptively and tested using the Student’s *t-*test or χ^2^ test. Survival was represented graphically using the Kaplan–Meier method and analysed using univariate and multivariate Cox regression. Hazard ratios (HRs) were represented graphically using forest plots. To match the baseline characteristics, we used propensity score matching. The propensity score was calculated by age, sex, presence of diabetes, medication (use of angiotensin-converting enzyme inhibitor, angiotensin receptor blocker, and erythropoiesis-stimulating agent), and laboratory data (haemoglobin, platelet, and estimated glomerular filtration rate (eGFR)) [21]. After propensity score matching, we used univariate and multivariate marginal structural Cox models for survival. The association between the use of sodium bicarbonate and heart failure symptoms at the initiation of dialysis therapy was assessed by univariate and multivariate logistic regression.

*P*-values of < 0.05 were considered significant. We used R (version 4.0.0, R Foundation for Statistical Computing, Vienna, Austria, URL http://www.R-project.org/) for all statistical analyses. For propensity score matching, the R package MatchIt (1:1 matching with the nearest neighbour with calliper matching, calliper = 0.1) was used for the calculation [22].

## Results

### Baseline characteristics

The patients’ baseline characteristics are shown in Table 1. The initial population included 1524 participants. Two patients who were untraceable were excluded. Among them, 1032 were men and 492 were women, with a mean age of 67.5 ± 13.1 years. Of the remaining 1522 patients, 677 used sodium bicarbonate and 845 did not. There was a significant difference between patients with and without sodium bicarbonate with regard to medical history or drug administration. Prevalence of past history, such as diabetes mellitus, heart failure, and stroke, was significantly lower in those who received sodium bicarbonate than in their counterparts (diabetes mellitus: 44.5% versus 56.3%, *p* < 0.001; admission of heart failure: 16.5% versus 23.7%, *p* = 0.001; stroke: 6.5% versus 11.0%, *p* = 0.003). The use of direct renin inhibitors, calcium channel blockers, angiotensin II receptor blockers or angiotensin-converting enzyme inhibitors, and vitamin D receptor agonist was significantly higher in patients on sodium bicarbonate than in their counterparts (direct renin inhibitor: 4.9% versus 2.5%, *p* = 0.018; calcium channel blocker: 84.0% versus 74.7%, *p* < 0.001; angiotensin II receptor blocker or angiotensin-converting enzyme inhibitors: 64.5% versus 57.1%, *p* = 0.004; vitamin-D receptor activator: 34.0% versus 21.5%, *p* < 0.001). Both serum pH and serum bicarbonate were significantly higher in those on sodium bicarbonate than in those without (pH: 7.35 ± 0.07 versus 7.33 ± 0.09, *p* < 0.001; bicarbonate: 20.24 ± 4.35 mEq/L versus 19.01 ± 5.33 mEq/L, *p* < 0.001). This may be because sodium bicarbonate reduces metabolic acidosis. eGFR was significantly lower in patients on sodium bicarbonate than in their counterparts (eGFR: 5.14 ± 1.85 mL/min/1.7m^2^ vs. 5.69 ± 2.45 mL/min/1.7m^2^, *p* < 0.001). Prevalence of all-cause mortality was significantly lower in patients on sodium bicarbonate than in their counterparts (13.6% versus 21.2%, *p* < 0.001).

**Table 1:** Baseline and clinical characteristics and outcomes of patients at the time of dialysis initiation between patients with sodium bicarbonate and without sodium bicarbonate (*n* = 1522)

	without sodium bicarbonate (*n* = 845)	with sodium bicarbonate (*n* = 677)	*p* value
Female (%)	278 (32.9)	214 (31.6)	0.632
Age (mean (SD))	67.74 (13.51)	67.20 (12.47)	0.420
Cause of CKD (%)			0.003
Diabetes Mellitus	396 (46.9)	262 (38.7)	
Nephrosclerosis	209 (24.7)	176 (26.0)	
Others, unknown	240 (28.4)	239 (35.3)	
Past history			
Diabetes Mellitus (%)	476 (56.3)	301 (44.5)	< 0.001
CAD (%)	152 (18.1)	104 (15.4)	0.186
PAD (%)	45 (5.3)	26 (3.8)	0.214
Atrial fibrillation (%)	61 (7.3)	33 (4.9)	0.073
Admission of HF (%)	200 (23.7)	112 (16.5)	0.001
Aortic Dissection (%)	50 (5.9)	34 (5.0)	0.514
Malignancy (%)	86 (10.2)	76 (11.2)	0.565
Stroke (%)	93 (11.0)	44 (6.5)	0.003
X-ray			
CTR (mean (SD))	55.58 (7.01)	54.68 (7.30)	0.015
Cardiac ultrasonography			
EF (mean (SD))	60.12 (12.74)	61.32 (11.73)	0.088
Administration			
Spironolactone (%)	42 (5.0)	34 (5.0)	1.000
DRI (%)	21 (2.5)	33 (4.9)	0.018
CCB (%)	631 (74.7)	569 (84.0)	< 0.001
Loop (%)	549 (65.0)	451 (66.6)	0.536
Thiazide (%)	208 (24.6)	140 (20.7)	0.079
ARBACEI (%)	481 (57.1)	437 (64.5)	0.004
BB (%)	302 (35.7)	226 (33.4)	0.365
Statin (%)	330 (39.1)	279 (41.2)	0.423
VDRA (%)	182 (21.5)	230 (34.0)	< 0.001
Anti Platelet (%)	274 (32.4)	186 (27.5)	0.042
ESA (%)	662 (78.6)	643 (95.0)	< 0.001
Laboratory data			
WBC (/uL) (mean (SD))	7113 (3493)	6302 (2604)	< 0.001
Hb (g/dL) (mean (SD))	9.26 (1.65)	9.52 (1.39)	0.001
Plt (10,000/uL) (mean (SD))	18.76 (8.33)	17.58 (6.65)	0.003
Alb (g/dL) (mean (SD))	3.15 (0.59)	3.27 (0.60)	< 0.001
BUN (mg/dL) (mean (SD))	94.00 (32.84)	88.99 (26.94)	0.001
Cr (mg/dL) (mean (SD))	8.75 (3.39)	9.25 (2.96)	0.002
eGFR (mL/min/1.73m^2^) (mean (SD))	5.69 (2.45)	5.14 (1.85)	< 0.001
Na (mEq/L) (mean (SD))	137.7 (4.4)	138.1 (4.3)	0.078
K (mEq/L) (mean (SD))	4.57 (0.88)	4.53 (0.78)	0.388
Adjusted Ca (mg/dL) (mean (SD))	8.69 (1.06)	8.52 (1.05)	0.002
P (mg/dL) (mean (SD))	6.43 (2.00)	6.29 (1.71)	0.145
Mg (mg/dL) (mean (SD))	2.17 (0.50)	2.13 (0.46)	0.206
UA (mg/dL) (mean (SD))	9.00 (2.57)	8.53 (2.23)	< 0.001
LDL-C (mg/dL) (mean (SD))	90 (35)	90 (34)	0.952
CRP (mg/dL) (mean (SD))	1.97 (4.09)	1.69 (4.25)	0.217
β2MG (ug/dL) (mean (SD))	19.29 (5.66)	19.15 (5.87)	0.730
TSAT (%) (mean (SD))	27.93 (17.55)	26.10 (15.54)	0.057
Ferritin (ng/dL) (median [IQR])	136.00 [71.00, 248.00]	112.70 [57.00, 202.25]	< 0.001
pH (mean (SD))	7.33 (0.09)	7.35 (0.07)	< 0.001
HCO_3_^−^ (mEq/L) (mean (SD))	19.01 (5.33)	20.24 (4.35)	< 0.001
Other			
HF symptoms at admission (%)	291 (34.6)	176 (26.0)	< 0.001
Outcome			
CVD-related death (%)	67 (8.0)	35 (5.2)	0.041
Infection-related death (%)	36 (4.3)	20 (3.0)	0.227
All-cause death (%)	179 (21.2)	92 (13.6)	< 0.001

*ARBACEI* angiotensin receptor blocker or angiotensin-converting enzyme inhibitor, *Adjusted Ca*; adjusted calcium, *Alb* albumin, *BB* beta blocker, *BUN* blood urea nitrogen, *β2MG* beta-2 microglobulin. *CAD* coronary artery disease, *CCB* calcium channel blocker, *CKD* chronic kidney disease, *Cr* creatinine, *CRP* C-reactive protein, *CTR* cardiothoracic ratio, *CVD* cardiovascular disease, *DRI* direct renin inhibitor, *EF* ejection fraction, *eGFR* estimated glomerular filtration rate, *ESA* erythropoietin stimulating agent, *Hb* hemoglobin, *HCO*_*3*_^*−*^ bicarbonate, *HF* heart failure, *IQR* interquartile range, *K* potassium, *LDL-C* low-density lipoprotein cholesterol, *Mg* magnesium, *Na* sodium, *P* phosphate, *PAD* peripheral arterial disease, *Plt* platelet, *SD* standard deviation, *TSAT* transferrin saturation, *UA* uric acid, *VDRA* vitamin D receptor agonist, *WBC* white blood cells

### Mortality

**Fig. 2:**
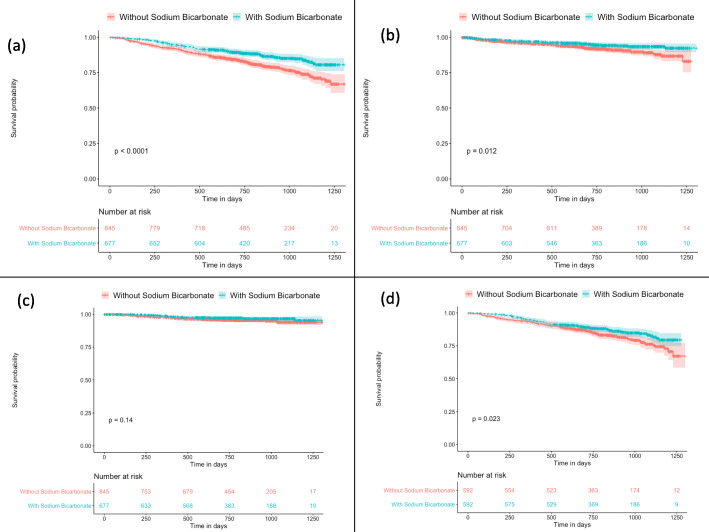
**a**: Kaplan–Meier plot for all-cause death with and without the use of oral sodium bicarbonate before dialysis initiation. **b**: Kaplan–Meier plot for CVD-related death with and without the use of oral sodium bicarbonate before dialysis initiation. **c**: Kaplan–Meier plot for infection-related death with and without the use of oral sodium bicarbonate before dialysis initiation. **d**: Kaplan–Meier plot for all-cause death in propensity score-matched patients with and without the use of oral sodium bicarbonate before dialysis initiation. CVD; cardiovascular disease

**Fig. 3:**
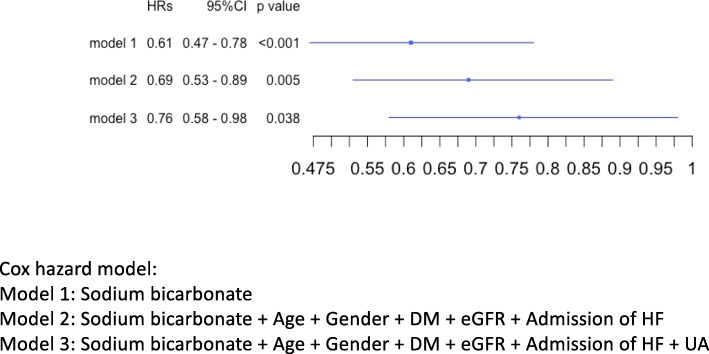
HRs of using oral sodium bicarbonate for all-cause death. HR; hazard ratio. CI; confidence interval. DM; diabetes mellitus. eGFR; estimated glomerular filtration rate. HF; heart failure. UA; uric acid

The median follow-up period was 814.5 days. During the follow-up period, 271 patients died from various causes, such as cardiovascular events (102 patients, 37.6%), infectious diseases (56 patients, 20.7%), cancer (45 patients, 16.6%), and other causes (68 patients, 25.1%). Figure 2(a) shows the Kaplan–Meier plot for all-cause mortality in patients with and without sodium bicarbonate therapy: the former group had a significantly lower mortality rate than the latter group (13.6% [92 patients] versus 21.2% [179 patients], *p* < 0.001) (Table 1). Figure 2(b) shows the Kaplan–Meier plot for cardiovascular disease (CVD) related mortality in patients with and without sodium bicarbonate. The former group had a significantly better prognosis than the latter group (*p* = 0.012). Figure 2(c) shows the Kaplan–Meier plot for infection-related mortality in patients with and without sodium bicarbonate. There was no significant difference between the two groups (*p* = 0.14). Figure 3 shows the forest plot for HRs of sodium bicarbonate for all-cause death with adjustment for various factors. Adjustment for various cofactors revealed that the use of sodium bicarbonate was independently related to better prognosis (HR, 0.76; 95% confidence interval, 0.58–0.98; *p* = 0.038).

### Associations between the use of sodium bicarbonate and heart failure symptoms

Table 2 shows the associations between the use of sodium bicarbonate and heart failure symptoms at the initiation of dialysis therapy by univariate and multivariate logistic regression. Even after adjustment, the use of sodium bicarbonate at dialysis initiation was not positively associated with heart failure symptoms at the initiation of dialysis therapy (adjusted odds ratio = 0.79, 95% confidence interval (CI) = 0.63–0.99, *p* < 0.05).

**Table 2:** Associations between use of sodium bicarbonate and heart failure symptoms at initiation of dialysis therapy

	Crude OR (95% CI)	Adjusted OR (95% CI)	p value
Without Sodium Bicarbonate	Reference	Reference	Reference
With Sodium Bicarbonate	0.66 (0.53–0.83)	0.79 (0.63–0.99)	< 0.05

*OR* odds ratio, *CI* confidence interval; other factors included diabetes mellitus, estimated glomerular filtration rate, calcium channel blocker, angiotensin-converting enzyme inhibitor and angiotensin receptor blocker

### Propensity score-matched comparison between patients with and without sodium bicarbonate therapy

**Fig. 4:**
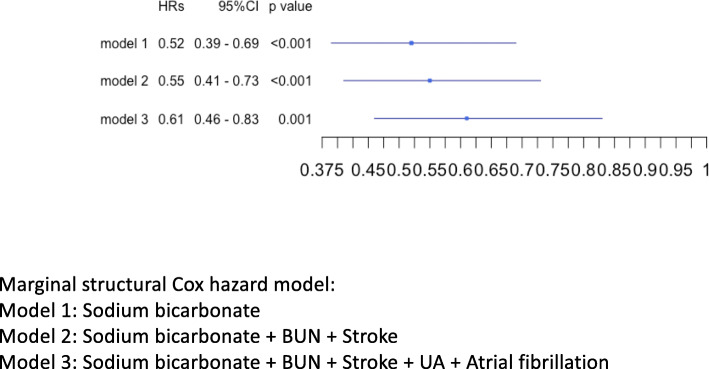
HRs by marginal structural Cox hazard model of using oral sodium bicarbonate for all-cause death in matched patients. HR; hazard ratio. BUN; blood urea nitrogen. UA; uric acid. VDRA; vitamin D receptor activator

The baseline and clinical characteristics in Table 1 showed significant differences between patients in the two groups, suggesting a possibility of bias. Table 3 shows the baseline characteristics of propensity score-matched patients with and without sodium bicarbonate. Regarding the patients’ background, there were still significant differences in atrial fibrillation, stroke, blood urea nitrogen, uric acid, ferritin, pH, bicarbonate, and the use of vitamin D receptor agonists. Figure 2(d) shows the Kaplan–Meier plot for all-cause death in matched patients with and without sodium bicarbonate (*p* = 0.023). Patients with sodium bicarbonate showed a significantly better prognosis than those without. There was no significant difference between matched patients with and without sodium bicarbonate in either CVD-related or infection-related death individually (Supplementary Fig. 1 and Supplementary Fig. 2). Figure 4 shows the forest plot for HRs of matched patients with sodium bicarbonate for all-cause mortality with adjustment for various factors. Adjustment for various cofactors revealed that the use of sodium bicarbonate could independently reduce mortality (HR, 0.61; 95% CI, 0.46–0.83; *p* = 0.001).

**Table 3:** Baseline and clinical characteristics and outcomes of propensity-score matched patients at the time of dialysis initiation between patients with sodium bicarbonate and without sodium bicarbonate (*n* = 1184)

	without sodium bicarbonate (*n* = 592)	with sodium bicarbonate (*n* = 592)	*p* value
Female (%)	185 (31.2)	193 (32.6)	0.663
Age (mean (SD))	67.93 (13.54)	67.53 (12.25)	0.592
Cause of CKD (%)			0.149
Diabetes Mellitus	275 (46.5)	256 (43.2)	
Nephrosclerosis	150 (25.3)	138 (23.3)	
Others, unknown	167 (28.2)	198 (33.4)	
Past history			
Diabetes Mellitus (%)	309 (52.2)	291 (49.2)	0.323
CAD (%)	102 (17.3)	93 (15.7)	0.506
PAD (%)	35 (5.9)	24 (4.1)	0.182
Atrial fibrillation (%)	46 (7.8)	26 (4.4)	0.020
Admission of HF (%)	125 (21.1)	103 (17.4)	0.122
Aortic Dissection (%)	35 (5.9)	29 (4.9)	0.516
Malignancy (%)	64 (10.8)	71 (12.0)	0.583
Stroke (%)	70 (11.8)	41 (6.9)	0.005
X-ray			
CTR (mean (SD))	55.14 (7.02)	54.95 (7.31)	0.648
Cardiac ultrasonography			
EF (mean (SD))	61.18 (11.75)	61.44 (11.79)	0.734
Administration			
Spironolactone (%)	24 (4.1)	33 (5.6)	0.277
DRI (%)	20 (3.4)	31 (5.2)	0.152
CCB (%)	472 (79.7)	498 (84.1)	0.059
Loop (%)	417 (70.4)	396 (66.9)	0.210
Thiazide (%)	151 (25.5)	122 (20.6)	0.053
ARBACEI (%)	366 (61.8)	370 (62.5)	0.857
BB (%)	220 (37.2)	199 (33.6)	0.224
Statin (%)	238 (40.2)	250 (42.2)	0.516
VDRA (%)	143 (24.2)	204 (34.5)	< 0.001
Anti Platelet (%)	197 (33.3)	168 (28.4)	0.078
ESA (%)	557 (94.1)	558 (94.3)	1.000
Laboratory data			
WBC (/uL) (mean (SD))	6555 (2711)	6344 (2607)	0.173
Hb (g/dL) (mean (SD))	9.42 (1.52)	9.46 (1.41)	0.638
Plt (10,000/uL) (mean (SD))	18.00 (7.29)	17.84 (6.70)	0.706
Alb (g/dL) (mean (SD))	3.20 (0.58)	3.24 (0.60)	0.203
BUN (mg/dL) (mean (SD))	94.56 (29.33)	88.00 (27.03)	< 0.001
Cr (mg/dL) (mean (SD))	8.92 (2.93)	9.08 (2.89)	0.346
eGFR (mL/min/1.73m^2^) (mean (SD))	5.34 (1.74)	5.22 (1.90)	0.289
Na (mEq/L) (mean (SD))	137.9 (4.2)	138.1 (4.4)	0.546
K (mEq/L) (mean (SD))	4.51 (0.83)	4.53 (0.80)	0.784
Adjusted Ca (mg/dL) (mean (SD))	8.67 (1.02)	8.56 (1.04)	0.064
P (mg/dL) (mean (SD))	6.38 (1.89)	6.24 (1.70)	0.174
Mg (mg/dL) (mean (SD))	2.18 (0.48)	2.14 (0.46)	0.177
UA (mg/dL) (mean (SD))	9.05 (2.39)	8.50 (2.19)	< 0.001
LDL-C (mg/dL) (mean (SD))	88 (32)	90 (35)	0.241
CRP (mg/dL) (mean (SD))	1.54 (3.67)	1.75 (4.41)	0.397
β2MG (ug/dL) (mean (SD))	19.37 (5.58)	19.15 (5.84)	0.623
TSAT (%) (mean (SD))	27.73 (16.77)	26.15 (15.72)	0.136
Ferritin (ng/dL) (median [IQR])	129.00 [68.00, 227.25]	112.00 [56.15, 197.50]	0.006
pH (mean (SD))	7.34 (0.08)	7.35 (0.07)	0.012
HCO_3_^−^ (mEql/L) (mean (SD))	19.46 (5.07)	20.37 (4.36)	0.003
Other			
HF symptoms at admission (%)	168 (28.6)	165 (27.9)	0.840
Outcome			
CVD-related death (%)	35 (6.0)	31 (5.3)	0.714
infection-related death (%)	25 (4.2)	17 (2.9)	0.271
All-cause death (%)	112 (18.9)	83 (14.0)	0.028

*ARBACEI* angiotensin receptor blocker or angiotensin-converting enzyme inhibitor, *Adjusted Ca* adjusted calcium, *Alb* albumin, *BB* beta blocker, *BUN* blood urea nitrogen, *β2MG* beta-2 microglobulin, *CAD* coronary artery disease, *CCB* calcium channel blocker, *CKD* chronic kidney disease, *Cr* creatinine, *CRP* C-reactive protein, *CTR* cardiothoracic ratio, *CVD* cardiovascular disease, *DRI* direct renin inhibitor, *EF* ejection fraction, *eGFR* estimated glomerular filtration rate, *ESA* erythropoietin stimulating agent, *Hb* hemoglobin, *HCO3*^*−*^ bicarbonate, *HF* heart failure, *IQR* interquartile range, *K* potassium, *LDL-C* low-density lipoprotein cholesterol, *Mg* magnesium, *Na* sodium, *P* phosphate, *PAD* peripheral arterial disease, *Plt* platelet, *SD* standard deviation, *TSAT* transferrin saturation, *UA* uric acid, *VDRA* vitamin D receptor agonist, *WBC* white blood cells

## Discussion

The results of the present study showed that patients who received sodium bicarbonate had a significantly better prognosis than those who did not. After propensity score matching, the trend remained similar. Moreover, we observed that the use of sodium bicarbonate did not increase the prevalence of heart failure symptoms at dialysis initiation.

Previously, the effect of oral alkali has been discussed [3, 11–13]. We observed significant differences in pH and bicarbonate levels with and without administration of sodium bicarbonate. This could reflect that oral sodium bicarbonate improved acid-base balance in patients with CKD before dialysis initiation. Moreover, we showed that the use of sodium bicarbonate even before dialysis therapy was significantly associated with a better prognosis in patients being initiated on dialysis therapy. There have been few reports regarding sodium bicarbonate and mortality in patients with ESKD [16]. As dialysis therapy can correct acid-base balance, it is important for clinicians to know how much impact the use of sodium bicarbonate before dialysis initiation has on the prognosis after dialysis initiation. We showed that the use of sodium bicarbonate before dialysis initiation could improve the prognosis of patients on dialysis therapy.

Our results show that oral sodium bicarbonate significantly reduced all-cause mortality, which is similar to a previous meta-analysis [13]. Although infection-related mortality was not independently associated with the use of oral sodium bicarbonate, CVD-related death was significantly associated with the use of sodium bicarbonate in the Kaplan–Meier plot before propensity score matching. It has been reported that acidosis contributes to bone demineralization [23]. Although there is controversy about the association between metabolic acidosis and ectopic calcification, we might observe a potential negative impact on ectopic vascular calcification due to metabolic acidosis. In contrast, after propensity score matching, there was no association between CVD-related death and sodium bicarbonate. Because we matched patients by age, diabetes mellitus, use of angiotensin-converting enzyme or angiotensin receptor blocker, the impact of vascular damage could be reduced in the matched model. We analysed associations between the use of sodium bicarbonate and heart failure symptoms at the initiation of dialysis therapy. The use of oral sodium bicarbonate was negatively associated with heart failure symptoms. Previously, it was hypothesised that sodium bicarbonate could worsen heart failure [3]. Our results contradict this hypothesis, probably because physicians prescribed sodium bicarbonate to those who were less likely to have heart failure. Our results are novel because the use of oral sodium bicarbonate before dialysis initiation did not increase the symptoms of heart failure at the time of dialysis initiation.

Our study has some strengths. First, our study included a well-defined population. Furthermore, our study had an extremely high follow-up rate. Second, we focused solely on the relationships between the use of oral sodium bicarbonate and mortality after dialysis initiation. Our study has some limitations. First, as this was a retrospective observational study, there is an inevitable selection bias regarding administration of sodium bicarbonate. By using multivariate Cox Hazard model, propensity score matching, even though we managed to reduce the selection bias, we cannot deny the potential bias. Second, the dosage, forms and total period of sodium bicarbonate administration in patients before dialysis initiation are unknown.

Because our study showed the importance of oral sodium bicarbonate in patients with CKD before dialysis initiation, a prospective randomized control study of oral sodium bicarbonate before dialysis initiation is required in the future. Furthermore, in our study, it was unclear how long period patients used oral sodium bicarbonate before dialysis initiation. Therefore, it is necessary to study when to begin using oral sodium bicarbonate and the appropriate control of pH.

## Conclusion

Patients on oral sodium bicarbonate at the time of dialysis initiation showed a better prognosis than those who did not receive sodium bicarbonate. Therefore, the use of sodium bicarbonate can be useful in patients with CKD who have acidosis.

## Supplementary Information

**Additional file 1.**

## Data Availability

The datasets used and/or analyzed during the current study are available from the corresponding author upon reasonable request.

## Abbreviations

AICOPP: Aichi Cohort Study of Prognosis in Patients Newly Initiated into Dialysis (AICOPP)
BUN: Blood Urea Nitrogen
CKD: Chronic kidney disease
CI: Confidence interval
DM: Diabetes mellitus
eGFR: Estimated glomerular filtration rate
ESKD: End-stage kidney disease
HF: Heart failure
HR: Hazard ratios
UA: Uric acid
VDRA: Vitamin D receptor agonist

## References

[1] Chen TK, Knicely DH, Grams ME (2019). Chronic kidney disease diagnosis and management: a review. JAMA..

[2] Webster AC, Nagler EV, Morton RL, Masson P (2017). Chronic kidney disease. Lancet..

[3] BiCARB study group (2020). Clinical and cost-effectiveness of oral sodium bicarbonate therapy for older patients with chronic kidney disease and low-grade acidosis (BiCARB): a pragmatic randomised, double-blind, placebo-controlled trial. BMC Med.

[4] Shah SN, Abramowitz M, Hostetter TH, Melamed ML (2009). Serum bicarbonate levels and the progression of kidney disease: a cohort study. Am J Kidney Dis.

[5] Dobre M, Yang W, Chen J, Drawz P, Hamm LL, Horwitz E, Hostetter T, Jaar B, Lora CM, Nessel L, Ojo A, Scialla J, Steigerwalt S, Teal V, Wolf M, Rahman M, CRIC Investigators (2013). Association of serum bicarbonate with risk of renal and cardiovascular outcomes in CKD: a report from the chronic renal insufficiency cohort (CRIC) study. Am J Kidney Dis.

[6] Raphael KL, Wei G, Baird BC, Greene T, Beddhu S (2011). Higher serum bicarbonate levels within the normal range are associated with better survival and renal outcomes in African Americans. Kidney Int.

[7] Tangri N, Stevens LA, Griffith J, Tighiouart H, Djurdjev O, Naimark D, Levin A, Levey AS (2011). A predictive model for progression of chronic kidney disease to kidney failure. JAMA..

[8] Menon V, Tighiouart H, Vaughn NS, Beck GJ, Kusek JW, Collins AJ, Greene T, Sarnak MJ (2010). Serum bicarbonate and long-term outcomes in CKD. Am J Kidney Dis.

[9] Kovesdy CP, Anderson JE, Kalantar-Zadeh K (2009). Association of serum bicarbonate levels with mortality in patients with non-dialysis-dependent CKD. Nephrol Dial Transplant.

[10] Raphael KL, Zhang Y, Wei G, Greene T, Cheung AK, Beddhu S (2013). Serum bicarbonate and mortality in adults in NHANES III. Nephrol Dial Transplant.

[11] de Brito-Ashurst I, Varagunam M, Raftery MJ, Yaqoob MM (2009). Bicarbonate supplementation slows progression of CKD and improves nutritional status. J Am Soc Nephrol.

[12] Phisitkul S, Khanna A, Simoni J, Broglio K, Sheather S, Rajab MH (2010). Amelioration of metabolic acidosis in patients with low GFR reduced kidney endothelin production and kidney injury, and better preserved GFR. Kidney Int.

[13] Susantitaphong P, Sewaralthahab K, Balk EM, Jaber BL, Madias NE (2012). Short- and long-term effects of alkali therapy in chronic kidney disease: a systematic review. Am J Nephrol.

[14] Vassalotti JA, Centor R, Turner BJ, Greer RC, Choi M, Sequist TD, et al. Practical approach to detection and management of chronic kidney disease for the primary care clinician. Am J Med. 2016; 129(2): 153–162.e7. doi:10.1016/j.amjmed.2015.08.025. 10.1016/j.amjmed.2015.08.025 26391748

[15] Abramowitz MK (2017). Bicarbonate balance and prescription in ESRD. J Am Soc Nephrol.

[16] Liu XY, Gao XM, Zhang N, Chen R, Wu F, Tao XC, Li CJ, Zhang P, Yu P (2017). Oral bicarbonate slows decline of residual renal function in peritoneal dialysis patients. Kidney Blood Press Res.

[17] Hu MK, Witham MD, Soiza RL. Oral bicarbonate therapy in non-haemodialysis dependent chronic kidney disease patients: a systematic review and meta-analysis of randomised controlled trials. J Clin Med. 2019; 8(2): 208. Published. doi:10.3390/jcm8020208. 10.3390/jcm8020208 PMC6406285 30736428

[18] Hishida M, Tamai H, Morinaga T, Maekawa M, Aoki T, Tomida H, Komatsu S, Kamiya T, Maruyama S, Matsuo S, Inaguma D (2016). Aichi cohort study of the prognosis in patients newly initiated into dialysis (AICOPP): baseline characteristics and trends observed in diabetic nephropathy. Clin Exp Nephrol.

[19] Tanaka A, Inaguma D, Shinjo H, Murata M, Takeda A (2016). Aichi cohort study of prognosis in patients newly initiated into Dialysis (AICOPP) study group. Presence of atrial fibrillation at the time of dialysis initiation is associated with mortality and cardiovascular events. Nephron.

[20] McKee PA, Castelli WP, McNamara PM, Kannel WB (1971). The natural history of congestive heart failure: the Framingham study. N Engl J Med.

[21] Shahbaz H, Gupta M. Creatinine clearance. In: *Stat*Pearls [Internet]. Treasure Island (FL): StatPearls Publishing. 2020 2 Jan–. PMID: 31334948:2020. 31334948

[22] Ho DE, Imai K, King G, Stuart EA (2011). MatchIt: nonparametric preprocessing for parametric causal inference. J Stat Soft.

[23] Raphael KL (2018). Metabolic acidosis and subclinical metabolic acidosis in CKD. J Am Soc Nephrol.

