# Supplementary material for: Relationship between mortality and use of sodium bicarbonate at the time of dialysis initiation: a prospective observational study
Source: BMC Nephrol. 2021 Apr 6;22:118. doi: 10.1186/s12882-021-02330-0 (PMC8025509; doi:10.1186/s12882-021-02330-0)

Relationship between mortality and use of sodium bicarbonate at the time of dialysis initiation: a prospective observational study

Hikaru Morooka,[1] Junichiro Yamamoto[2], Akihito Tanaka,[1] Daijo Inaguma[3], Shoichi Maruyama[4]

[1]Division of Nephrology, Nagoya University Hospital, Nagoya, Japan

[2] Division of Nephrology, Tsushima City Hospital, Tsushima, Japan

[3]Division of Internal Medicine, Fujita Health University Bantane Hospital, Nagoya, Japan

[4]Division of Nephrology, Nagoya University Graduate School of Medicine, Nagoya, Japan

Corresponding author: Akihito Tanaka

Division of Nephrology,

Nagoya University Hospital

Tsurumaicho, 65, Showa Ward, Nagoya, Aichi, Japan

Tel: +81-52-741-2111

Fax: +81-52-744-2209

E-mail: tanaka17@med.nagoya-u.ac.jp

Supplemental material

Supplementary figure 1: Kaplan-Meier plot for CVD-related death in matched patients with and without use of oral sodium bicarbonate before dialysis initiation. CVD; cardiovascular disease.

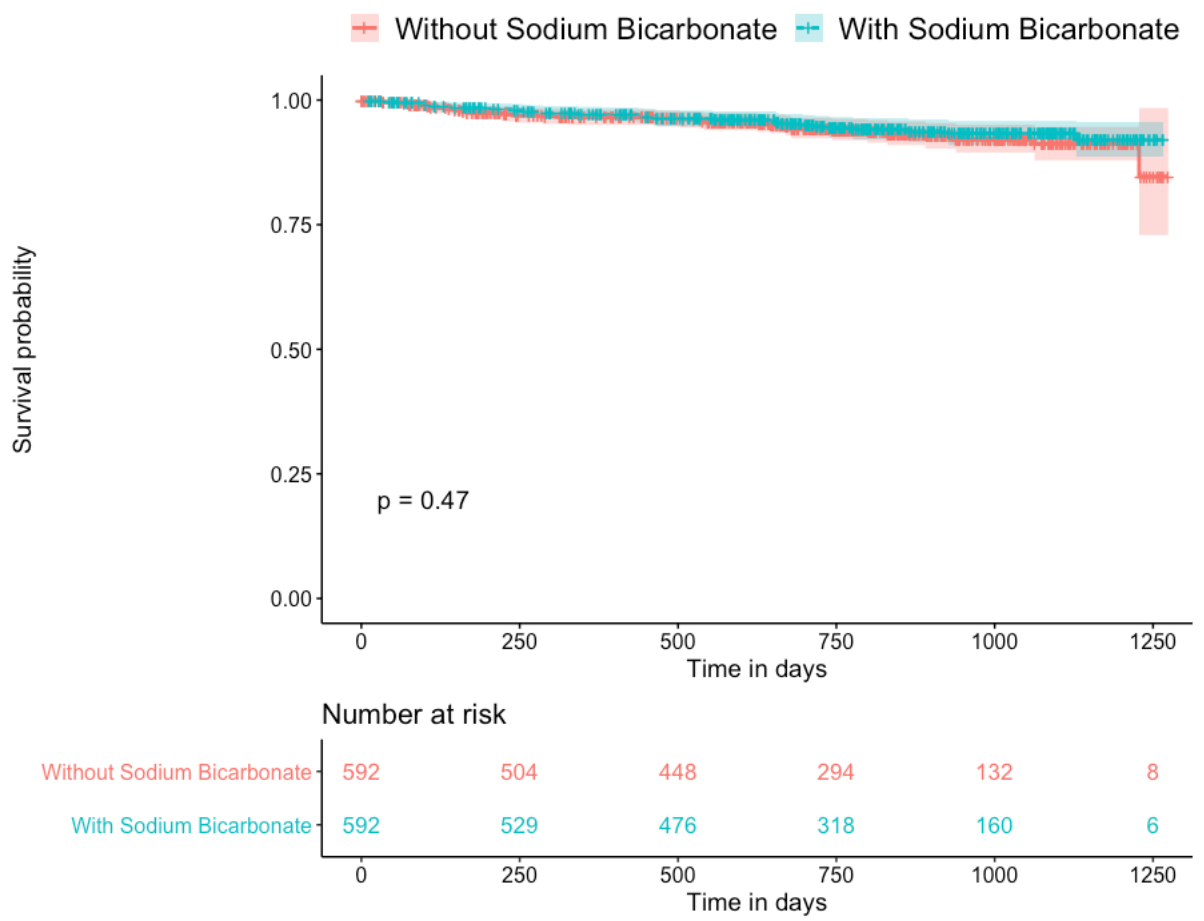

Supplementary figure 2: Kaplan-Meier plot for infection-related death in matched patients with and without use of oral sodium bicarbonate before dialysis initiation.

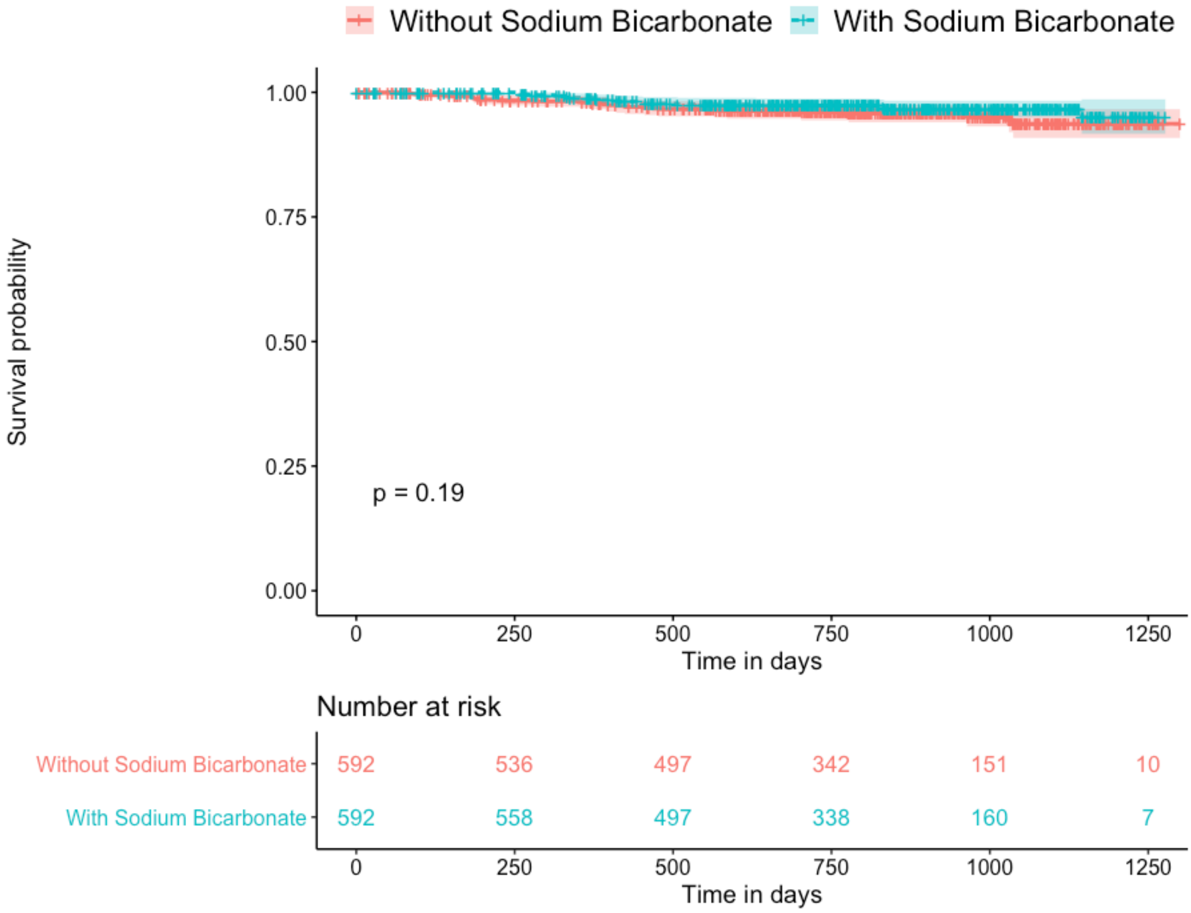

Supplement: Supplementary file 1 — Additional file 1. [file 12882_2021_2330_MOESM1_ESM.pdf]
